# Mitigating Phototoxicity during Multiphoton Microscopy of Live *Drosophila* Embryos in the 1.0–1.2 *µ*m Wavelength Range

**DOI:** 10.1371/journal.pone.0104250

**Published:** 2014-08-11

**Authors:** Delphine Débarre, Nicolas Olivier, Willy Supatto, Emmanuel Beaurepaire

**Affiliations:** 1 Laboratory for Optics and Biosciences, Ecole Polytechnique, CNRS UMR 7645, and INSERM U696, Palaiseau, France; 2 Univ. Grenoble Alpes, LIPhy, Grenoble, France; 3 CNRS, LIPhy, Grenoble, France; Glasgow University, United Kingdom

## Abstract

Light-induced toxicity is a fundamental bottleneck in microscopic imaging of live embryos. In this article, after a review of photodamage mechanisms in cells and tissues, we assess photo-perturbation under illumination conditions relevant for point-scanning multiphoton imaging of live *Drosophila* embryos. We use third-harmonic generation (THG) imaging of developmental processes in embryos excited by pulsed near-infrared light in the 1.0–1.2 *µ*m range. We study the influence of imaging rate, wavelength, and pulse duration on the short-term and long-term perturbation of development and define criteria for safe imaging. We show that under illumination conditions typical for multiphoton imaging, photodamage in this system arises through 2- and/or 3-photon absorption processes and in a cumulative manner. Based on this analysis, we derive general guidelines for improving the signal-to-damage ratio in two-photon (2PEF/SHG) or THG imaging by adjusting the pulse duration and/or the imaging rate. Finally, we report label-free time-lapse 3D THG imaging of gastrulating *Drosophila* embryos with sampling appropriate for the visualisation of morphogenetic movements in wild-type and mutant embryos, and long-term multiharmonic (THG-SHG) imaging of development until hatching.

## Introduction

Avoiding light-induced toxicity is a fundamental and poorly understood bottleneck in long-term microscopic imaging of live embryos. For example, although live *Drosophila* or zebrafish embryos can be observed with subcellular three-dimensional (3D) resolution using confocal microscopy, photodamage limits the temporal and spatial resolution that can be achieved without perturbing their development. One efficient way to circumvent this issue is to use an imaging technique where light absorption occurs mostly in the imaging plane, such as selective plane illumination microscopy (SPIM) [1] or multiphoton microscopy [2]. In particular, multiphoton (or nonlinear) microscopy uses excitation wavelengths in the near-infrared (NIR) range (700–1300 nm), and is attractive for embryo studies because it combines a good penetration depth and a limited perturbation of development compared to confocal microscopy, as shown in [3]. This latter property has been attributed to the reduced one-photon (or linear) absorption of cell components in the NIR range.

Nevertheless, nonlinear microscopy requires illumination with tightly focused femtosecond laser pulses, *i.e.* illumination intensities that can reach several hundreds of GW/cm^2^. This illumination regime is close to conditions causing local destructive effects by nonlinear light-tissue interactions [4], [5]. It is therefore of critical importance to investigate the influence of illumination parameters on embryonic tissue and to define guidelines for safe imaging.

Third-harmonic generation (THG) microscopy [6], [7] is a nonlinear microscopy technique that has recently gained interest for recording structural images of unstained biological samples. Excitation is usually performed in the 1.0–1.5 *µ*m range, and signal is detected at 1/3 of this wavelength. In particular, THG imaging has been used to quantify morphogenetic movements in gastrulating *Drosophila* embryos [8], to reconstruct the cell lineage in early zebrafish embryos [9], to visualise *C. elegans* embryogenesis [10], and to assess the development and viability of mammalian embryos [11], [12]. Being a label-free imaging modality, it is well-suited for assessing perturbations induced by pulsed infrared light during multiphoton imaging.

Since little is known about the mechanisms of phototoxicity in the 1.0–1.2 *µ*m range, we use in this study THG imaging to assess the perturbation of *Drosophila* embryo development induced by femtosecond pulsed illumination, as a function of relevant imaging parameters such as excitation wavelength in the 1.0–1.2 *µ*m range, pulse duration and time between successive images. Using both short-term and long-term indicators of development perturbations, we investigate the origin of the observed photodamage. In particular and in agreement with previous studies at shorter wavelengths, we find that photodamage depends on the excitation intensity in a supra-quadratic manner (exponent greater than 2), because these tissues do not possess strong one-photon absorbers such as pigments. Finally, we establish guidelines for safe nonlinear imaging of live embryos.

## Review of Photodamage Mechanisms

**Figure 1:**
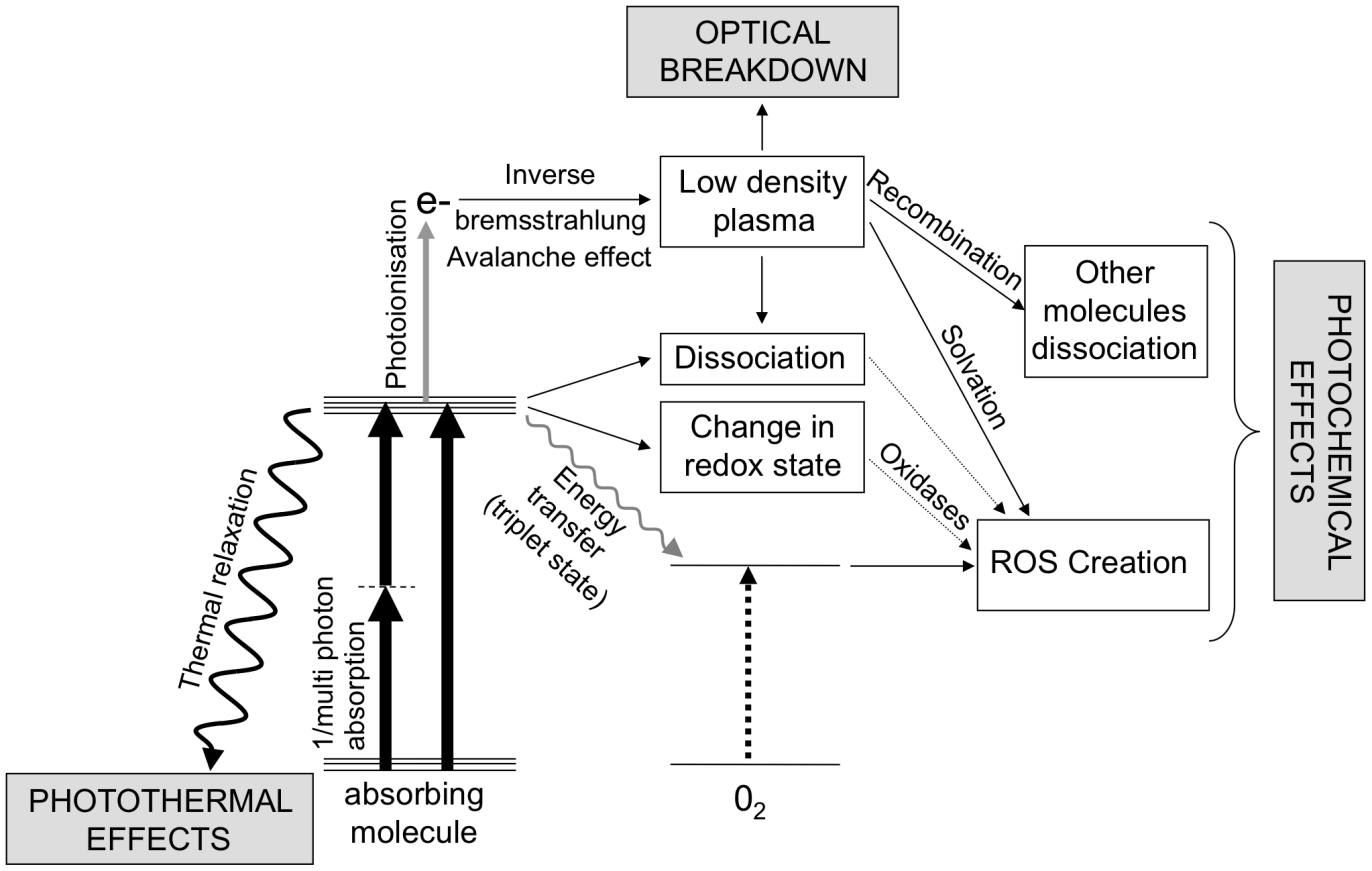
Mechanisms of light-induced cell perturbations. Summary of the main processes involved at the onset of photoperturbation during near infrared imaging, as discussed in [13], [14], [43]. See text for more details.

In order to analyse the origin of the damage observed in our experiments, we first present a brief summary of the various photo-perturbation effects described in the literature. A wealth of studies has been published on the effects of light on biological tissues, which correspond to very diverse illumination conditions. However, despite major experimental differences in terms of excitation wavelength, illumination time or pulse duration, the described effects share common features and can all be included in a general diagram (figure 1). In short, four main types of experimental conditions have been studied:

- Illumination by a weakly focused, continuous light source with moderate average power (typical intensities of a few to tens of W/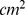): this type of scheme, with wavelengths ranging from the near ultraviolet (UV) to the near IR, is used in medical contexts, either to induce cell proliferation (low level light therapy, LLLT), or on the contrary to destroy cells with the assistance of a photosensitizer (photodynamics therapy, PDT) [13]–[18]. Similar illumination conditions are also used in brightfield, phase contrast or conventional fluorescence imaging.
- Use of a tightly focused (NA = 1.0–1.4), continuous, high average power (50–200 mW) beam for optical trapping of organelles, cells or exogenous particules. In this case, the beam is generally stationary in one or several points of the cell [19]–[22].
- Use of a high average power (20–200 mW) pulsed infrared beam for local photodestruction of cellular or subcellular regions. In this case, the beam is either stationary within the cell or moved along a line [5], [23]–[32].
- Multiphoton microscopy, using high peak power (1–1000 GW/cm^2^), short (50–5000 fs) infrared (700–1300 nm) pulses at high repetition rates (80–120 MHz in most cases). In this case, the beam is scanned along two or three dimensions in order to form an image of the sample [3], [33]–[42].

In all cases, the interaction of the illumination light wave with the tissue involves the presence of absorbers. These primarily consist of water molecules or other tissue components, depending on the excitation wavelength. The first step of the photodamage process therefore consists in single-photon or multiphoton absorption events driving molecules to an excited state (figure 1). Several de-excitation pathways are then possible [43].

- First, the molecule can transfer its energy to the bulk in a non radiative manner. In this case, the tissue temperature is raised locally and illumination triggers thermal damage.
- Other mechanisms result in local chemical perturbations. This is the case for de-excitation paths which involve dissociation or change in the redox state of the absorbing molecule, or transfer of its energy to a dioxygen molecule to form reactive oxygen species (ROS).
- If the light intensity is high enough, ionisation of molecules occurs following additional (multi) photon absorption. Released electrons can then gain energy by absorbing photons during collisions with other molecules, a phenomenon known as the inverse bremsstrahlung effect [43]. Beyond a certain energy, free electrons can create additional free electrons during collisions. This avalanche effect leads to the creation of a low density plasma, which induces photochemical damage.
- Alternatively, if the optical breakdown threshold (typically 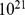 electrons/cm^3^) is reached, this plasma is no longer confined and triggers mechanical effects such as the formation of a supersonic shock wave, causing immediate destruction of local tissue structures and cell death [43]–[45]. Optical breakdown can be obtained under illumination conditions relatively close to those used in multiphoton imaging, e.g. by increasing the illumination intensity by one order of magnitude [40], [46]. It is accompanied by intense, broadband luminescence with short (1–5 ns) lifetime [40].

We note that optical breakdown is not relevant in the experiments described here, as the plasma density can be estimated to be several orders of magnitudes lower than the breakdown threshold (see table 1 and [43]). Similarly, photothermal effects can be discarded as a source of photodamage in typical multiphoton imaging conditions, unless the sample exhibits strong one-photon absorption (such as in the case of pigmented cells [35]). This assumption will be justified in the discussion. In the next paragraph, we will therefore restrict ourselves to the description of photochemical damage.

**Table 1:** Illumination parameters used in this work.

Excitation wavelength	1180 nm
Mean power at the focal point	120 mW
Pulse duration at the focal point	250 fs/100 fs
Repetition rate	76.4 MHz/80 MHz
Energy per pulse	1.57 nJ
Numerical aperture of the focusing objective	0.6–0.7
Mean intensity at the focal point	1.36 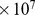 W/cm^2^
Peak intensity at the focal point	7.1 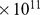 W/cm^2^
Scanning speed on the sample	150 *µ*m/ms
Time between 2 consecutive image lines	7.5 ms
Pixel size	0.6 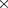 0.6 *µ*m^2^
Total image size	5 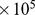 pixels
% of image occupied by the embryo	40–50% (depending on embryo)

Depending on the experimental parameters used in the cited studies, these various effects can arise from one, two or even three photon absorption by the tissue, and can be mediated to a various degree by the presence of a low density plasma (free electrons) [32]. They depend on the nature of the absorbing molecules involved, as their intracellular localisation will influence the type of perturbation. However, except in the case where UV light or <750 nm pulsed light is used and direct damage to DNA is induced, all studies report a perturbation of mitochondria and of the respiratory chain of the cell, even at low illumination intensities [47]. Indeed, mitochondria contain a high density of molecules that exhibit strong absorption in the visible or UV spectral range (cytochromes, flavoproteins, NAD(P)H, etc.) [48]. Different molecules may be primarily involved depending on the wavelength, but a following step generally involves the creation of ROS and the subsequent rise of oxidative stress [38]. This stress is then transducted differently depending on the amount of the perturbation [15], [49]: if perturbation is limited, repair and protection mechanisms take place that induce cell proliferation. This is the case in LLLT, but also in certain cases during multiphoton microscopy [33], [50]. However larger amounts of perturbation can exceed the repair capacity of the cell, and toxic effects gradually take place: increase in intracellular calcium concentration [15], [23], [39], membrane depolarisation [51], ROS diffusion inside the cytoplasm [14], destruction of nuclear membranes [37], breakdown of DNA synthesis [20], [49] and finally apoptotic cell death [37].

The induced effects are therefore a multi-parametric consequence of experimental illumination conditions, which explains why the reported threshold for photo-perturbation can seem very different from one study to the other. In fact, different studies use various damage criteria (morphological description, assessment of various cell functions, cell survival, etc.) as well as different illumination conditions (continuous or pulsed sources, stationary or scanned beam, focusing conditions, imaging time, etc.), and the parameters required for comparison are often lacking. As a result, although direct comparison cannot be conducted quantitatively, published studies provide us with a framework to analyze our results.

## Results: Influence of Illumination Parameters

Assessing phototoxicity in live organisms such as 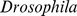 embryos is complex compared to the case of cultured cells where the use of probes for ROS production is straighfoward. Instead, we have chosen here to use two complementary criteria to evaluate photodamage levels.

**Figure 2:**
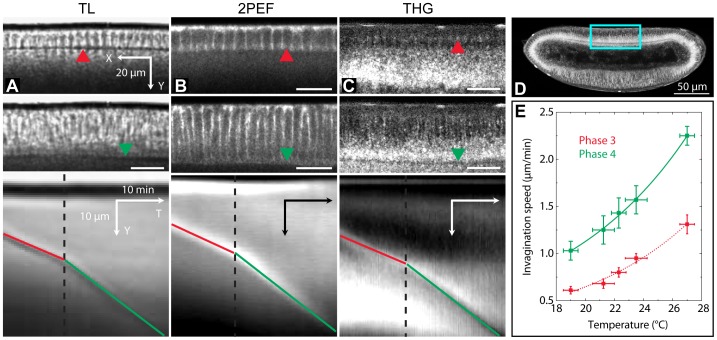
Assessing photoperturbation using THG imaging of cellularization dynamics in *Drosophila* embryos. Principle of cellular front invagination (CFI) speed measurement: (A), transmitted light imaging (wild-type embryo); (B), two-photon imaging (GFP-moesin-tagged embryo, outlining the cell boundaries); (C) and movie 1, THG imaging (wild-type embryo). The images in (A) – (C) are a zoom over the dorsal equatorial region of different embryos, corresponding approximately to the blue square in (D) on a THG image. Images (A) to (C) share the same scale bars. Top, phase 3 of cellularization; middle, phase 4 of cellularization; bottom, kymographs (YT projections) obtained from the time-lapse XY images, showing the propagation of the CFI over time. The dotted black time indicates the limit between phase 3 and phase 4, and the position of the CFI is indicated by a red (resp. green) line in phase 3 (resp. 4). Kymographs shown here as an example were obtained from time-lapse acquisitions with (A) 2 images/min; (B), 1 image/min; (C), 3 images/min. (E), CFI speed calibration as a function of temperature using transmitted light imaging. Errors bars are the standard deviations from 3 different embryos per temperature point.

- Long-term damage was assessed by monitoring embryo survival and its development into a larva (roughly 24 hours after cellularization at 20°C).
- In a complementary manner, we have used the dynamics of the cellularization process as a probe of short-term perturbation of development, and in particular of the integrity of the cytoskeleton (fig. 2): indeed, *Drosophila* embryos during the first stages of development consist of a multinuclear cell (syncytial blastoderm), and the cell membranes separating the nuclei invaginate towards the center of the embryo during cellularization (stage 5 of development as defined in [52]). Cellularization is used as a model for cell cytokinesis [53]. We have measured the speed (see section 6.3) of the cellularization front invagination (CFI) during the latest phases of cellularization (phases 3 and 4, as defined in [54]). First, the speed of CFI was measured using transmitted light microscopy as a function of temperature (figure 2(E), see [5] for more details). These values were used as a control in later experiments. Then, we verified that CFI measurements were consistent when imaging embryos using transmitted-light (TL), 2PEF, and THG. CFI speed measurements were then systematically conducted using the THG images acquired during illumination of unlabelled embryos. One example of typical images of the CFI and the resulting kymographs is shown on figure 2. On THG images the CFI contrast arises from the local change in the lipid droplet concentration: during phase 3, droplets accumulate around the CFI, giving rise to a positive contrast, whereas at a later stage, the CFI correspond to a depletion zone within a dense droplet region, thereby inducing a local decrease in the signal. Despite this change in contrast, the signal modulation ensures a precision of typically 0.1 *µ*m/min for the measurement of CFI speed.

In order to allow a comparison with other studies, we have listed in table 1 all the relevant illumination parameters. Unless otherwise mentioned, these are common to all the experiments presented in this paper, and the focal plane of the objective was matching the sagittal plane of the embryo. Finally, illumination was performed during cellularization and gastrulation (stage 5 to 8; see [52]), corresponding to a total duration of 90 minutes at 19°C.

### 3.1 Damage as a function of imaging rate

**Figure 3:**
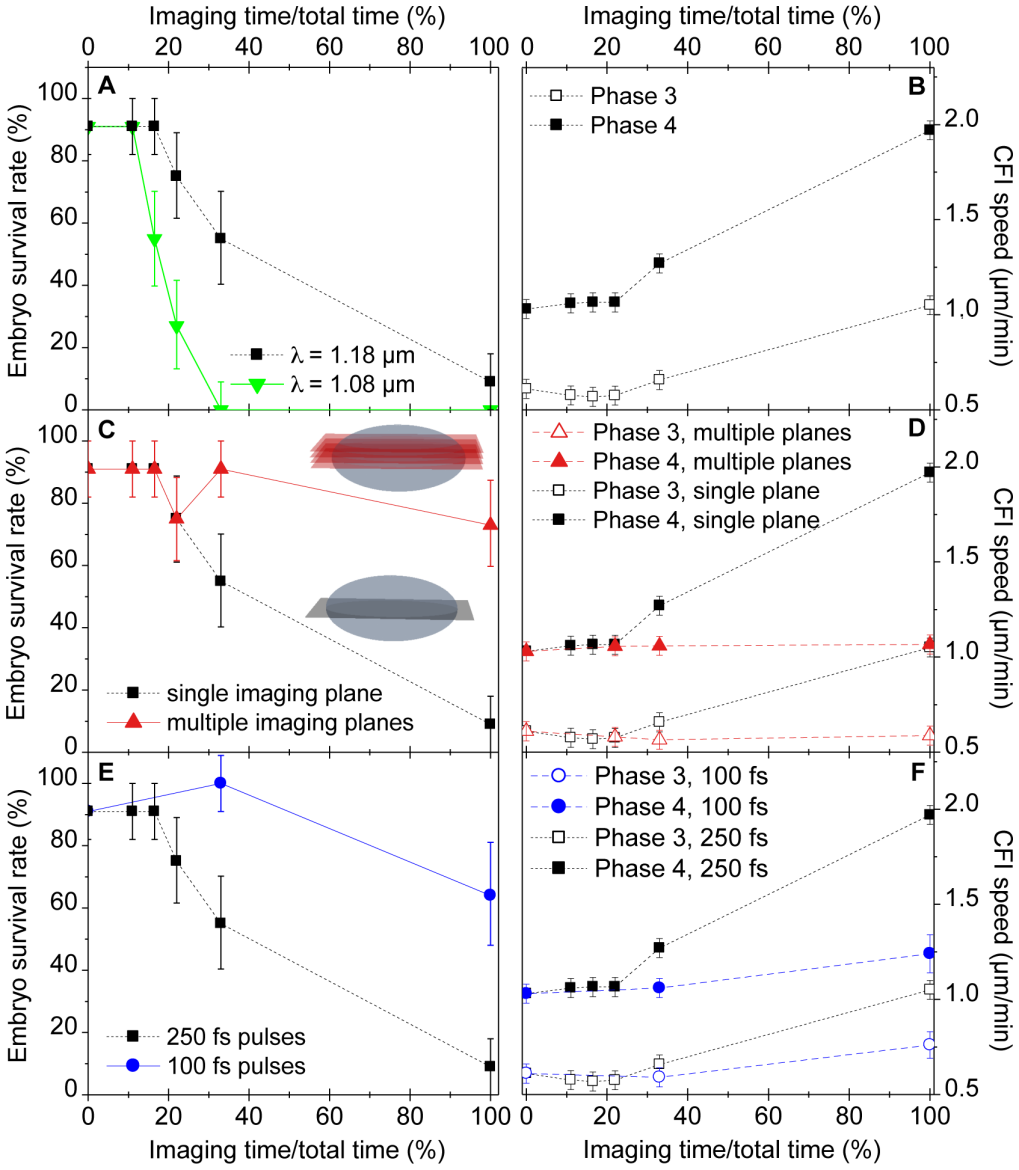
Influence of imaging parameters on light-induced perturbation during multiphoton imaging of *Drosophila* embryos at 1.18 *µ*m. (A,B) Cumulative effects and wavelength dependence. (A), embryo survival rate and (B), CFI speed (see figure 2) as a function of excitation wavelength and illumination rate. (C,D) Effect of the spatial spreading of the illuminated planes. (C) embryo survival rate, and (D) CFI rate, for volume (red) and single plane (black) imaging. Volume imaging was achieved by acquiring 2,3,4,6 or 18 images (3.2 s per image) axially separated by 2 *µ*m every 60 s, whereas for single plane imaging the same number of images where acquired always in the same plane (see cartoons in (C)). The survival rate is not significantly decreased when the embryo is continuously illuminated provided that the imaging rate in each plane is kept low (1 image/minute). (E,F) Influence of the pulse duration on development photoperturbation. THG efficiency, scaling as P^3^/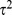, is kept constant. (E), embryo survival rate, and (F), CFI speed, as a function of imaging rate. All error bars are the standard deviation of the mean over 11 measurements for each data point.

**Figure 4:**
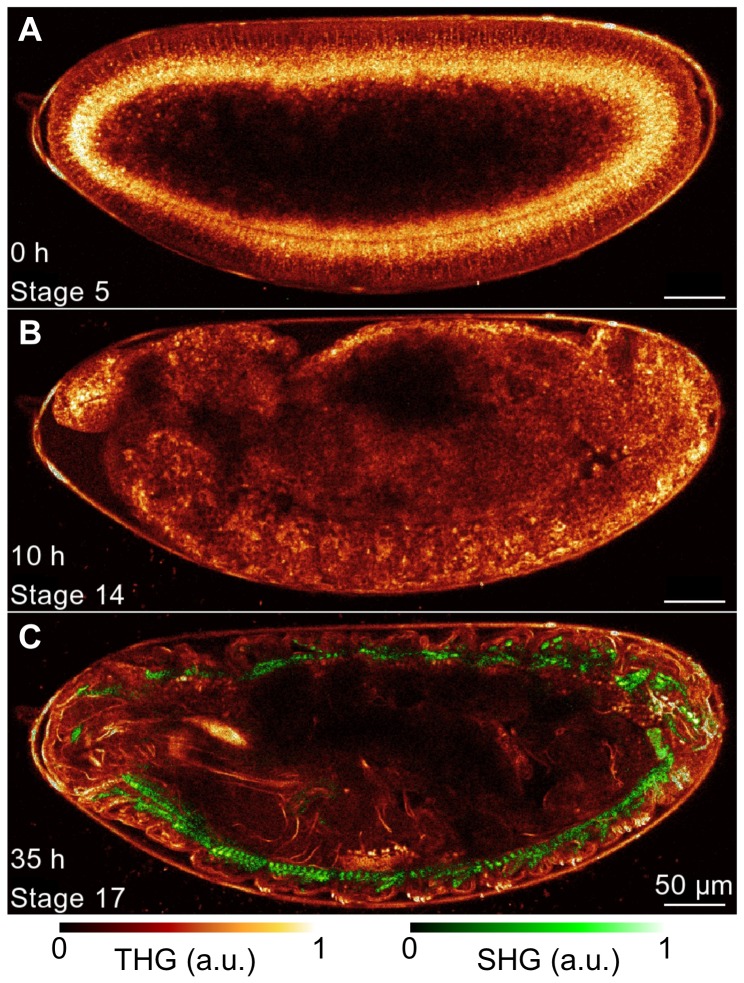
Long-term THG-SHG imaging of *Drosophila* embryonic development. 2D THG-SHG imaging at a wavelength of 1180 nm of a wild-type *Drosophila* embryo during 36 hours starting from stage 5 up to the larvae stage (i.e. until hatching) and imaged during 3.3 s every 150 s, corresponding to an imaging rate of 2%. (A–C) representative 2D THG-SHG images at different stages of the development, with stage and time after the beginning of the acquisition mentioned in the bottom left. The look-up-table used to represent the SHG and THG signals are displayed below. Scale bar  = 50 *µ*m. See also movie 2 for the full dataset.

First, we studied the perturbation of development as a function of the time between two successive illuminations of the embryo. More precisely, we considered the effect of the percentage of time during which the embryo was illuminated throughout the time-lapse sequence acquisition (duty cycle, or imaging rate). The results are summarized on figure 3, (A) and (B). Above a given threshold (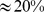 imaging rate in our conditions), the survival rate of the embryos drops, revealing significant perturbation of development (figure 3(A), black curve). Concurrently, increase in the speed of CFI both in phases 3 and 4 shows that the perturbation of development occurs within a few minutes after the start of the experiment, and can lead to CFI speeds twice as high as in unperturbed embryos (figure 3(B)). However, when the embryo is illuminated at a sustainable rate, i.e. below the perturbation threshold, its development can be imaged entirely up to the larva stage, as demonstrated using simultaneous THG-SHG imaging on figure 4 and Video S2. This movie was recorded with an imaging rate of 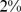 (imaging time being 3.3s per image). The total imaging time was 36 hours, which corresponds to over a thousand exposures, and the total illumination dose was comparable to that obtained for continuous exposure of the embryo during developmental stage 5 to 8. Nevertheless, we did not observe morphological perturbation or delay in development, indicating that organism-level damage arises beyond a given imaging rate and not simply beyond a given illumination dose.

### 3.2 Damage as a function of wavelength

As a second step, we investigated the influence of the excitation wavelength on the perturbation threshold. One possible origin of the damage is heating through one-photon absorption of the excitation light by the sample. At such early stage of development, the embryo does not exhibit significant pigmentation (melanin, etc.) that, if present, could significantly contribute to one-photon absorption in the near-infrared range [55]. The main one-photon absorber in this wavelength range is therefore water. We thus shifted the excitation wavelength from 1.18 to 1.08 *µ*m (while keeping all other illumination parameters constant), corresponding to a drop of the absorption coefficient of water from 1.04 cm^−1^ to 0.13 cm^−1^ [56]. If there was damage induced by one-photon absorption of light by water molecules, its amplitude should also have dropped significantly in this experiment. However, as can be seen on figure 3(A) (green curve), we found that phototoxicity increased for a given illumination rate. This suggests that perturbation did not originate here from one-photon absorption by water, but rather from multiphoton absorption by other tissue components.

### 3.3 Single plane vs. multiple plane imaging

In order to further test the hypothesis that damage arises through multiphoton (rather than one-photon) absorption, we performed similar measurements of the perturbation of development, but this time using multiple plane imaging. Using 1.18 *µ*m excitation wavelength, we increased the number of images acquired per minute, spreading them over multiple imaging planes separated by 2 *µ*m in z, such that the last plane corresponded to the sagittal plane of the embryo whereas the other planes plane were located closer to the objective. For example, in the case of continuous illumination of the sample, 17 images were acquired and constituted a 32 *µ*m-thick three-dimensional image. The survival rates and cellularization speed extracted from these experiments are presented on figure 3(C–D). Contrary to the case where a single plane is imaged (black curve), the survival rate and the cellularization speed of the embryos imaged on multiple planes were not perturbed (red curve), even in the case of continuous exposure. This indicates that the perturbation is confined in three dimensions, so that less cumulative damage is induced in a particular region of the embryo when the illumination is scanned in three dimensions instead of two. This further supports the hypothesis that phototoxicity is mainly mediated through a nonlinear process such as two- or three-photon absorption rather than by one-photon absorption of water, pigments or other endogenous molecules in the embryo: indeed, one-photon absorption is not confined axially so that the same amount of the damage that it mediates is induced in all the planes perpendicular to the beam propagation in the embryo, irrespective of the position of the image plane itself. Therefore if one-photon absorption was responsible for the observed photodamage, the two curves in figure 3C would be superimposed.

### 3.4 Influence of pulse duration

Finally, we determined the order of the nonlinear absorption process inducing the damage, i.e. whether it is a two-photon or a higher-order process, by investigating the influence of the excitation pulses duration. To that aim, we shortened the pulse duration at the focus from 250 fs to 100 fs (see methods). Transmission losses in the prism setup simultaneously reduced the excitation power at the sample surface from 120 mW to 70 mW. Since THG scales as 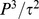, where 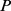 is the excitation power and 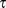 is the pulse duration, the THG signal was moderately increased by 25%. We then repeated the experiment described in paragraph 3.1, and illuminated embryos in a single plane with different imaging rates. The results are presented on figure 3(E–F), with the short pulse data represented in blue and the reference 250 fs data represented in black. From measured values of both survival rate and cellularization speed, it is clear that the phototoxicity is reduced when using shorter pulses. Since photodamage was reduced while THG (a third-order process) was slightly increased, and since we have previously ruled out one-photon absorption as a major source of photodamage in our experiments, this suggests that photodamage arises through a process of order lower than 3, e.g. through two-photon absorption or a mixture of two- and three-photon processes.

### 3.5 Label-free 3D imaging of gastrulating Drosophila embryos with THG

**Figure 5:**
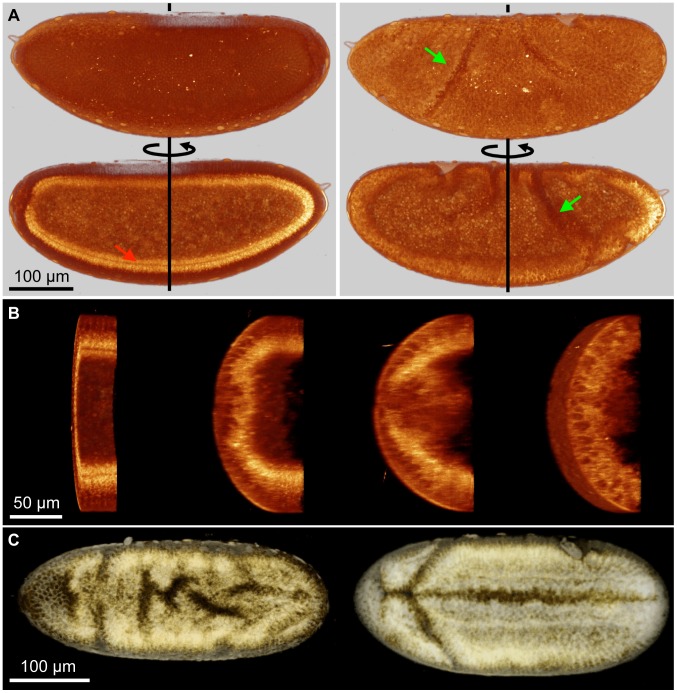
3D, label-free characterization of gastrulation movements in wild-type and mutant *Drosophila* embryos. (A) 3D dynamic visualisation of half a wild-type *Drosophila* embryo during gastrulation. Imaging conditions: 57 s per 3D stack, 2 min between successive stacks, 750 nm/pixel lateral sampling, 2 *µ*m/pixel axial sampling, 1180 nm excitation wavelength, 100 fs pulses. (A), sagittal view (through the ventral furrow). Left, cellularization (stage 5); right, gastrulation (stage 8). Red arrow, CFI. Green arrow, cephalic furrow. (B) and movie 3, ventral furrow formation visualised over a transverse slice of a half-embryo image acquired in successive frontal (coronal) planes. (C) THG imaging of the ventral side of Sna- (left) and wild-type (right) *Drosophila* embryos during gastrulation. Imaging conditions: 40 s per 3D stack, 750 nm/pixel lateral sampling, 3 *µ*m/pixel axial sampling, 3 *µ* *s*/pixel integration time, 30 planes by stack, continuous imaging, 1180 nm excitation wavelength, 100 fs pulses. See also movie 4.

From these data, we established a set of parameters for safe THG imaging of 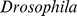 embryos during cellularization and gastrulation (figure 4, figure 5). Under these conditions, morphogenetic movements can be observed in 3D over time, as shown in figure 5 where the imaging rate is 50% spread over 30 planes (see figure 3(C) red curves for phototoxicity curve in comparable conditions but with a longer pulse width). Here, tissue deformations during gastrulation can be observed in a label-free manner with subcellular resolution over a volume containing half of the embryo, and with a time resolution of 2 minutes. 3D reconstructions permits characterizing the formation of structures such as the cephalic furrow (figure 5(A)) and the ventral furrow (figure 5(B)).

This 3D+t label-free imaging ability is of particular interest for comparing the dynamic phenotypes of wild-type and mutant embryos, as demonstrated in figure 5(C) and movie 4. In this experiment, continuous THG imaging is used to provide a dynamic 3D view of the ventral side a 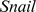 mutant embryo (sna-, left) and of a wild-type embryo (right) with a time resolution of 40 s. 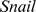 is known to be involved in the mesoderm invagination process [57], and mutation of this gene results in the disruption of ventral furrow formation. The images further reveal the existence of uncoordinated ventral cell movements and limited invagination in the muntant (sna-) phenotype. This example illustrates the potential of THG imaging for comparing the developmental dynamics of wild type and mutant phenotypes.

## Discussion

### 4.1 Tolerable illumination rates

From these results, some characteristics of the photodamage induced during multiphoton imaging of live *Drosophila* embryos can be deduced. First, the study of the impact of the rate of illumination on photodamage indicates that development perturbation occurs above a given threshold (figure 3). This suggests that the biological response to light irradiation arises from a competition between the previously described damage mechanisms and relaxation mechanisms, such as diffusion of toxic species or heat out of the focal plane, or repair mechanisms. As a result, biological perturbation occurs when the rate of photodamage exceeds the recovering ability of the sample, at the scale of the processes being observed. Furthermore, the parameters that were probed in this study show a relatively abrupt threshold between illumination conditions for which no or little perturbation is observed and conditions for which strong changes in the development appear. Such threshold is also evidenced by the large reduction of the observed perturbation when modifying the pulse duration and average excitation power, while two-photon excitation efficiency is only reduced by 25%.

Surprisingly, photoperturbation results in an increase in the CFI speed in both phase 3 and 4. Although one could instead expect a decrease in speed, evidences of counter-intuitive response to illumination stress are documented in other contexts such as LLLT (see review above). Such an increase in the CFI speed has also been reported near photoablated regions [4].

Below the damage threshold defined by the change in CFI rate and in survival rate, we have not observed residual developmental defects such as altered cell movements [58]: indeed, THG velocimetric analyses of well-described morphogenetic movements such as germ-band extension show normal displacement rates [8] using similar imaging parameters, and in the experiments reported here we did not observe developmental delays up to the larval stage in illuminated embryos. Complementary experiments using intracellular ROS probes [59] might detect more subtle effects but are difficult to implement on developing embryos without introducing preparation artifacts.

This observation that phototoxicity occurs only beyond a particular imaging rate can be related to the study of Squirrel et al. [3] on the long term development of hamster embryos after two-photon imaging at 1047 nm. Although direct quantitative comparison is difficult, it should be noted that the peak intensity in their experiment is only about 2.5 times smaller than the one used here, with an exposure time about 3 times longer and a shorter excitation wavelength - which we showed induces more damage (see figure 3). In these conditions, Squirrel *et al.* did not observe any decrease in the embryo long term survival rate nor any significant production of ROS. Although care should be taken when comparing our results with results obtained on mammalian embryos, this hints that ROS production is probably limited in our experiments.

A handful of other studies have used THG microscopy for imaging embryo development. In particular, zebrafish [9], [60], [61] and mouse [11], [12], [62] embryos have been imaged at various development stages, and several assessments of related phototoxicity have been conducted. Results are, again, difficult to quantitatively compare with the present study as both the sample and the imaging parameters are different, with some information lacking in the articles (e.g. pulse duration). Some studies [12], [60], [61] proposed that multiphoton imaging at around 1.23 *µ*m is innocuous, based on the survival rates of zebrafish and mouse embryos after continuous imaging for 12 hours (zebrafish) and 10 minutes (mouse). Other studies however reported measurable photo-perturbation in zebrafish (intracellular THG signal increase) [9] and in mouse embryos (drop in survival rate after implantation) [11], [62]. The latter studies, in particular, were conducted with imaging parameters very similar to ours (1.23 *µ*m excitation wavelength, 0.9–1.0 NA, 8 *µ*s pixel dwell time - see articles for more details) but with a laser power about 4 times lower, and concluded that the effects of illumination in mouse embryos are strongly dependent on the embryonic stage. Indeed, they found that continuous illumination over a reduced period of time was less detrimental to embryo development than sparse, long-term (12 h) imaging. This is in contrast with our findings, and suggests that perturbation is likely stage and tissue-specific. However, although biological response might differ for various organisms, the imaging parameters that we identify as playing a key role in the onset of photodamage can be expected to play a similar role: in the absence of pigmentation, for example, the influence of the pulse width and peak intensity are expected to influence development of other embryos in a similar way. Repeated illumination of the same plane in the embryo can be expected to play a significant role as well. The guidelines derived here can thus be useful to optimize multiphoton imaging in other systems.

### 4.2 Mechanisms of photodamage during multiphoton imaging in the 1.0–1.2 *µ*m range

Our data provide hints on the mechanisms of photodamage in developing embryos when using pulsed NIR excitation. First, the dependence of photodamage on the excitation wavelength (figure 3(A)) shows that one-photon absorption by water is not a dominant perturbation factor in point-scanning multiphoton imaging, even at 1.18 *µ*m excitation. This is in agreement with calculations by Schönle *et al.* [63] and Vogel *et al.* [43]: using their model and the parameters from Table 1, we find that the transient heating during the 6.7 *µ*s that is required to scan the beam over a surface of 1 *µ*m^2^ is comprised between 0.4°C [63] and 0.9°C [43]. This increase in temperature is well below the activation threshold for heat shock proteins [22] and is sufficiently small to be entirely resorbed between two successive image lines (the typical relaxation time in pure water is of the order of a few 100 *µ*s [21]). Since *Drosophila* embryos develop normally at temperatures in the range 17–30°C, it can be expected that such a limited rise in temperature will not significantly influence development. Indeed in the literature, thermal damage has been reported mostly in the case of the use of pulsed YAG lasers (for which the temperature increase can reach 100°C [64]), in the case of continuous YAG lasers such as used for optical trapping [21], [22], or in the case of strong absorption of the excitation wavelength by endogenous molecules such as pigments [35], [65].

Optical breakdown effects can also be ruled out in the illumination conditions used in this study. In contrast with what is observed after illuminating cells in the 750–900 nm range [33], [66], [67], we have never observed increases in autofluorescence (excited at 800 nm) after irradiating embryos with 1.1–1.2 *µ*m pulsed light. This is in agreement with results from a previous study peformed at 1.23 *µ*m [34]. The possible formation of a low-density plasma in the tissue that would contribute to the observed photochemical damage can not be ruled out. However, our data suggests that the main effects in our imaging conditions derive from multiphoton absorption by endogenous molecules. The large reduction of phototoxic effects observed when shortening the excitation pulses at constant THG signal level indicates that two-photon processes play a major role in these photo-perturbation, in agreement with previous studies at shorter wavelengths [36].

Although three-photon absorption (3PA) efficiency is generally less than that of two-photon absorption, 3PA might also contribute to tissue photo-sensitivity, e.g. when enhanced by an intermediate two-photon resonance. Photodamage might thus arise through a mixture of two- and three- photon absorption processes and therefore exhibit a complex dependence on the excitation intensity and pulse duration, e.g. scale as the intensity to the power 2.5. Such a dependence has indeed been reported by [39] when exciting around 800 nm with moderate powers (20–50 mW), as well as by our group in the case of femtosecond photo-ablation at 830 nm in *Drosophila* embryos [5], and more recently in the context of endogenous fluorescence imaging [41]. It should be noted, however, that the three-photon absorption cross-section is expected to drop significantly from 800 nm to 1.2 *µ*m (reflecting the drop of one-photon absorption from 250 to 400 nm and of two-photon absorption from 400 to 600 nm of most biological compounds, see e.g. http://omlc.ogi.edu/spectra/), so that the power dependence of phototoxic effects at 1.18 *µ*m might be closer to 2.

### 4.3 Limiting phototoxicity during multiphoton imaging of embryos

The 2-to-3 power dependence of perturbation on laser intensity in the wavelength range considered here dictates guidelines for optimizing live multiphoton imaging, depending on the contrast modality being used. When imaging unpigmented tissue with second-order processes such as 2PEF or SHG, reducing the excitation peak power (*e.g.* by lenghtening the pulse duration) is expected to reduce photobleaching and phototoxicity compared to signal level, in line with previous observations [68], [69]. This case corresponds to the most common situation in multiphoton microscopy. In contrast, when imaging with third-order processes such as THG or in the presence of strong one-photon absorbers in the laser path [35], [65], the signal-to-damage ratio is improved by using shorter, more intense pulses.

Furthermore, accumulation effects appear from our data to have a major effect on development perturbations. Further improvement in the reduction of phototoxic effects might thus be obtained by increasing the scanning speed on the sample [70] and adapting the scanning pattern to increase the delay between two successive illuminations on the same point, e.g. by using random scanning patterns [71]. At shorter time scales (1 ns–1 *µ*s), it has also been shown that reducing the repetition rate of the excitation source could significantly reduce photobleaching and photodamages [23], [72], [73]. The use of lower repetition rate sources might therefore also be beneficial, although it can be expected that additional higher order damage will arise if the peak excitation power is increased [43].

Finally, we have shown that shifting the excitation towards longer wavelengths is of critical importance to limit phototoxicity in the range explored here. This result is in agreement with previous studies [9], [11], [60]–[62] using 1.18–1.23 *µ*m, and might be explained by the drop in two- and three-photon absorption of many endogenous species, in particular flavins and NAD(P)H [74] which have been proposed to mediate the creation of ROS upon illumination [14], [15], [47], [75]. As a comparison, comparable excitation powers and focusing conditions have been shown in a previous study to lead to immediate tissue disruption through photoablation [5]. With the development of red and infrared fluorescent proteins and dyes [76]–[78], red-shifted wavelengths also permit the use of two-photon excited fluorescence, and have been shown to improve penetration depth [42], [79]. Since nonlinear susceptibility (and therefore THG efficiency) generally decreases with increasing wavelengths, following the trend of linear refractive indices [80], and since one-photon water absorption increases after 1.3 *µ*m, the 1.1–1.3 *µ*m range offers a very good compromise for THG imaging.

## Conclusion

In this study, we have used time-lapse THG images of developmental processes to assess the organism-level perturbation caused by pulsed illumination in the 1.1–1.2 *µ*m range in *Drosophila* embryos under conditions relevant for point-scanning multiphoton imaging. The analysis of the influence of illumination parameters on the short-term and long-term perturbation of development showed that photodamage primarily arise through 2- and/or 3-photon absorption processes and in a cumulative manner. As a consequence, the THG (resp. 2PEF-SHG) signal-to-damage ratio can be greatly improved by shortening (resp. lengthening) the excitation pulses, and by spacing out successive illuminations of the same plane in the tissue. By using these guidelines to optimize THG imaging, we have shown that label-free imaging of half a gastrulating *Drosophila* embryo is possible with sub-cellular spatial resolution and 40–120 s temporal sampling, enabling direct visualisation of morphogenetic movements in wild-type and mutant embryos.

## Material and Methods

### 6.1 Preparation of biological samples

Oregon-R (http://flybase.org/reports/FBsn0000005.html) was used as the wild-type *Drosophila melanogaster* strain. Embryos were collected at the onset of developmental stage 5 (stages defined in [52]), dechorionated with bleach, and glued to a coverslip as previously described [58]. During image acquisition, they were maintained in PBS at room temperature (19 +/−1°C). After imaging, the coverslip was sealed in a Petri dish to prevent PBS evaporation, and kept at room temperature for about 24 hours. After that time, transmitted light imaging was used to assess whether they had developed up to the larval stage. For figure 2, a sGMCA-GFP strain (Flybase FBtp0012637) [81] was used instead, and for figure 5(C), a Sna*^IIG^*/Cyo strain,Bloomington fly stock (Flybase FBal0015904).

Survival rate measurements: for each experiment, 10 to 15 embryos were dechorionated and glued to the same coverslip. One of them, correctly oriented and at the right stage, was illuminated, while the others served as control for assessing the survival rate 24 hours later, estimated from the hatching and the movement of the larva. Control embryos were glued several millimeters away from the illuminated embryo, thereby ensuring that they were not significantly illuminated during the imaging process. For each imaging condition (fixed illumination power, rate, pulse duration, etc.), 11 embryos were imaged in different experiments (1 per coverslip). The number of control embryos for each imaging condition was thus 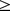
100.

### 6.2 Optical setup and imaging conditions

Multiphoton imaging was performed using a custom-built scanning microscope providing several contrast modalities including two-photon excited fluorescence (2PEF), second-harmonic and third-harmonic generation (SHG, THG). The microscope incorporated a femtosecond Titanium:Sapphire (Ti:S) laser (920 nm, 80 MHz, Mira, Coherent Inc., CA, USA) an optical parametric oscillator (1080–1180 nm, KTP-OPO, APE, Germany), galvanometric mirrors (GSI Lumonics, Poole, United Kingdom), a 0.95 NA 20x water-immersion objective (XLUMPFL, Olympus, Tokyo, Japan), photomultiplier modules (Electron Tubes, Ruislip, United Kingdom), and lab-designed counting electronics. Scanning and acquisition were synchronized using lab-written LabVIEW software and a multichannel I/O board (PCI-6115, National Instruments, USA).

For THG imaging, the diameter of the OPO excitation beam was set so as to underfill the back aperture of the objective to obtain an effective numerical aperture of 0.6–0.7 in order to optimize contrast in 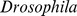 embryos [82]. The value of the effective numerical aperture was calculated based on the experimentally measured axial resolution from a THG signal profile at a glass coverslip/water interface perpendicular to the beam propagation. Simultaneous THG and SHG signals (figure 4) were detected in the forward direction and selected using appropriate dichroic and bandpass filters (495 dclp, 390/40, and 590/20, Semrock, Rochester, NY, USA). GFP 2PEF imaging (figure 2) was performed with 920 nm Ti:S excitation and epidetection of the fluorescence (HQ525/50, and 695CDXRU Chroma Technology Corp, USA).

The excitation parameters for THG imaging are summarized in Table 1. The same parameters were used throughout this study unless otherwise stated. In particular, all other parameters were kept constant when changing the excitation wavelength. Apart from the multi-plane imaging assay, imaging was always performed in a single plane at the equator of the embryo.

Pulse shortening (paragraph 4.4) was achieved using a pair of SF14 prisms in double-pass arrangement [83]. Pulse duration was measured at the focal spot of the microscope objective using an autocorrelator (pulseCheck, APE, Germany).

### 6.3 Data analysis

- 1D-kymograph analysis for monitoring the rate of cellularization front invagination (CFI) was performed using ImageJ (W. Rasband, National Institutes of Health, Bethesda, MD) according to [8]. Briefly, the XYT stacks of images of the relevant region of the embryo were projected along the X axis for improved signal-to-noise ratio, thereby obtaining YT images with Y axis in the direction of cellularization front progression showing the displacement of the CFI over time as a line whose slope is directly related to the CFI speed. This speed naturally varies during the progression of invagination (see figure 2), defining four phases with different rates [54]. Imaging was performed during phases 3 and 4 of CFI, and subsequent gastrulation. We therefore determined from these kymographs the CFI speed during these two phases. Speed was obtained by hand from the slope of the CFI on kymographs.
- 3D image reconstruction of developing embryos (figure 5) was performed using AMIRA (Mercury Computer Systems, Chelmsford, MA), while 2D rendering was obtained with ImageJ.

## Supporting Information

Video S1 **Example of kymograph construction from a time-lapse movie of the CFI from a developing** ***Drosophila*** **embryo imaged with THG.** CFI speed is measured on the kymograph as the slope of manually adjusted segments for phase 3 (red) and phase 4 (green). See also figure 2.
(AVI)

Video S2 **Long-term imaging of a developing** ***Drosophila*** **embryo with THG (red) and SHG (green).** Imaging starts at stage 5 (about 3 h post fertilization) until hatching of the larva 36 hours later. Imaging is performed with 1180 nm excitation during 3.3 s every 150 s. THG shows a structural image of the embryo, while SHG outlines the formation of the muscles. See also figure 4.
(AVI)

Video S3 **3D imaging of an unstained** ***Drosophila*** **embryo with THG during gastrulation (stages 5–8).** Imaging conditions: 57 s per 3D stack, 2 min between successive stacks, 750 nm per pixel lateral sampling, 2 *µ*m per pixel axial sampling, 1180 nm excitation wavelength, 100 fs pulses. 3D THG imaging allows the monitoring of the ventral furrow formation. Scale bar  = 100 *µ*m. See also figure 5.
(AVI)

Video S4 **3D imaging of a sna- (top) and a wild-type (bottom)** ***Drosophila*** **embryo with THG during gastrulation (stages 5–8).** Imaging conditions: 750 nm per pixel lateral sampling, 3 *µ*m per pixel axial sampling, 3 *µ* *s* per pixel, 30 planes by stack, 40 s per stack, continuous imaging, 1180 nm excitation wavelength, 100 fs pulses. Such dataset permits a direct comparison of morphogenetic movements in wild-type and mutant embryos for which fluorescent strains are often lacking. See also figure 5.
(AVI)
